# Functional Amyloid Protection in the Eye Lens: Retention of α-Crystallin Molecular Chaperone Activity after Modification into Amyloid Fibrils

**DOI:** 10.3390/biom7030067

**Published:** 2017-09-12

**Authors:** Megan Garvey, Heath Ecroyd, Nicholas J. Ray, Juliet A. Gerrard, John A. Carver

**Affiliations:** 1 CSL Limited, 45 Poplar Road, Parkville, VIC 3052, Australia; 2 School of Biological Sciences and the Illawarra Health and Medical Research Institute, University of Wollongong, Wollongong, NSW 2522, Australia; heathe@uow.edu.au; 3 Research School of Chemistry, The Australian National University, Acton, ACT 2601, Australia; nicholas.ray@anu.edu.au (N.J.R.); john.carver@anu.edu.au (J.A.C.); 4 School of Biological Science and School of Chemical Science, University of Auckland, Auckland 1010, New Zealand; j.gerrard@auckland.ac.nz

**Keywords:** amyloid fibril, small heat-shock protein, molecular chaperone, protein unfolding, protein aggregation

## Abstract

Amyloid fibril formation occurs from a wide range of peptides and proteins and is typically associated with a loss of protein function and/or a gain of toxic function, as the native structure of the protein undergoes major alteration to form a cross β-sheet array. It is now well recognised that some amyloid fibrils have a biological function, which has led to increased interest in the potential that these so-called functional amyloids may either retain the function of the native protein, or gain function upon adopting a fibrillar structure. Herein, we investigate the molecular chaperone ability of α-crystallin, the predominant eye lens protein which is composed of two related subunits αA- and αB-crystallin, and its capacity to retain and even enhance its chaperone activity after forming aggregate structures under conditions of thermal and chemical stress. We demonstrate that both eye lens α-crystallin and αB-crystallin (which is also found extensively outside the lens) retain, to a significant degree, their molecular chaperone activity under conditions of structural change, including after formation into amyloid fibrils and amorphous aggregates. The results can be related directly to the effects of aging on the structure and chaperone function of α-crystallin in the eye lens, particularly its ability to prevent crystallin protein aggregation and hence lens opacification associated with cataract formation.

## 1. Introduction

The crystallin proteins are primarily found within the mammalian eye lens where they form part of the protein array that focuses light onto the retina via a supramolecular, liquid-like order [1,2,3]. Crystallins are highly stable proteins, as there is very limited protein turnover in the lens, particularly in its centre [4,5]. α-crystallin, the predominant protein of the human lens, is a heterogeneous oligomer comprised of two closely related subunits, αA- and αB-crystallin, in a ratio of approximately 3:1, respectively [6]. α-crystallin oligomers are composed of between 15 and 50 of these subunits and have an average mass of approximately 700 kDa [7,8]. The two α-crystallin subunits are members of the small heat-shock protein (sHsp) family of molecular chaperone proteins. In the eye lens, their chaperone action is important in maintaining the stability and solubility of other crystallin proteins. αB-crystallin (HSPB5), but not αA-crystallin (HSPB4), is also found in many other tissues where it performs a variety of roles in protein stabilisation [9,10,11].

The identification of αB-crystallin in amyloid fibril deposits associated with a number of neurodegenerative diseases, e.g., Alzheimer’s and Parkinson’s, suggests that the protein may play a role in their progression by acting to prevent protein aggregation leading to amyloid fibril formation [9,10,12,13]. As a result, significant research activity has been undertaken into the mechanism by which αA- and αB-crystallin act as chaperones, both as homo- and hetero-oligomers. Delineation of the means by which sHsps act as chaperones could have significant potential therapeutically.

Chaperone activity is proposed to involve mainly hydrophobic interactions between the accessible hydrophobic region(s) on the chaperone and the exposed hydrophobic core of the unfolding target protein [14,15,16]. The exact role hydrophobicity plays in chaperone-related interactions between amorphously aggregating target proteins and α-crystallin is unclear. Potential chaperone binding sites have been identified for αA- and αB-crystallin [17,18,19,20,21,22]. The interaction and complexation of these chaperones with partially folded target proteins acts to stabilize the target protein, preventing its aggregation and precipitation and, in vivo, potentially staves off disease states that arise due to unfolding and mis-folding of such proteins.

As implied from their name, sHsp expression is elevated under conditions of thermal cellular stress [23]. At elevated temperatures, the structure of α-crystallin is altered due to partial unfolding and a subsequent enhancement in chaperone activity occurs. αA-crystallin in particular has increased chaperone efficiency at elevated temperature [24,25] which is postulated to be linked to an increase in exposed hydrophobicity at temperatures above 50 °C [6]. αB-rystallin also retains its ability to act as a chaperone at temperatures above 60 °C [24,25]. Thermal stress is not the only situation that may lead to increased chaperone activity of α-crystallin. Das and Liang [26] demonstrated that α-crystallin, when unfolded by guanidine hydrochloride (GdnHCl), forms a molten globule (intermediate) state with enhanced chaperone ability. They argued that such intermediate forms of α-crystallin would occur in vivo due to post-translational modifications, such as glycation, oxidation and mixed disulfide formation. From the above, it is apparent that αA- and αB-crystallin act effectively as molecular chaperones under conditions of cellular stress (i.e., during heat shock) even when their structure is disrupted or partially unfolded [27,28].

Concomitant with α-crystallin’s thermally enhanced chaperone ability is an increase in size of the α-crystallin oligomer, i.e., the diameter of the α-crystallin oligomer increases from 14.7 ± 2.7 nm at 35 °C to 26.8 ± 4.3 nm at 60 °C [29]. The enhanced chaperone activity is proposed to be associated with increased hydrophobicity of α-crystallin under thermal stress, rather than to be linked to oligomer size [16]. An increase in subunit exchange is also proposed to be a factor in the enhanced chaperone activity of α-crystallin at higher temperature [18]. The ability of urea-denatured α-crystallin to refold into smaller oligomers that exhibit an increased chaperone activity is consistent with these factors [30]. However, cross-linked α-crystallin retains chaperone activity [31,32], implying that the dissociated (probably dimeric) form of α-crystallin is not entirely responsible for the chaperone action of the protein, i.e., the oligomeric form retains at least some chaperone activity.

In contrast to amorphous aggregation, amyloid fibril formation results in the protein being rearranged into a well-ordered cross β-sheet fibrillar structure [33,34]. Amyloid fibrils are frequently associated with cellular damage in vivo, due to the formation of toxic precursors and the deposition of large conglomerates known as amyloid plaques composed primarily of amyloid fibrils [35]. Amyloid fibrils are a structure potentially accessible to all proteins and can be formed in vitro from proteins not associated with disease [36]. As well as being highly ordered, amyloid fibrils are thermodynamically stable and are resistant to proteolysis, dehydration and extremes of pH, temperature or pressure [37,38,39,40]. The identification of biologically active amyloid species, known as functional amyloid, has increased the interest in the potential uses of such a stable core structure of proteins for a wide variety of applications [36,41,42,43].

Protein aggregation and sHsp chaperone activity can be studied in vitro under a variety of conditions related to the properties of the aggregating protein and the type of aggregation it undergoes (either amorphous or amyloid fibrillar) [22,44]. Herein, a range of target proteins and destabilisation techniques were used, providing a comprehensive picture of α-crystallin chaperone activity in its various aggregation states, i.e., the native, amorphous and amyloid fibrillar forms. The results provide insights into the role aggregated α-crystallin plays in both the ageing eye lens, where the formation of crystallin aggregates leads to lens opacification and cataract, and in extra-lenticular disorders such as Alzheimer’s and Parkinson’s diseases and other amyloidosis, which are associated with the formation of amyloid fibrillar aggregates.

## 2. Results

Nomenclature: In this work, α-crystallin purified from bovine lenses (i.e., containing both αA- and αB-crystallin subunits) is referred to as α-crystallin, in order to delineate it from recombinant human αB-crystallin.

### 2.1. α-Crystallin Amyloid Fibril Formation

**Figure 1:**
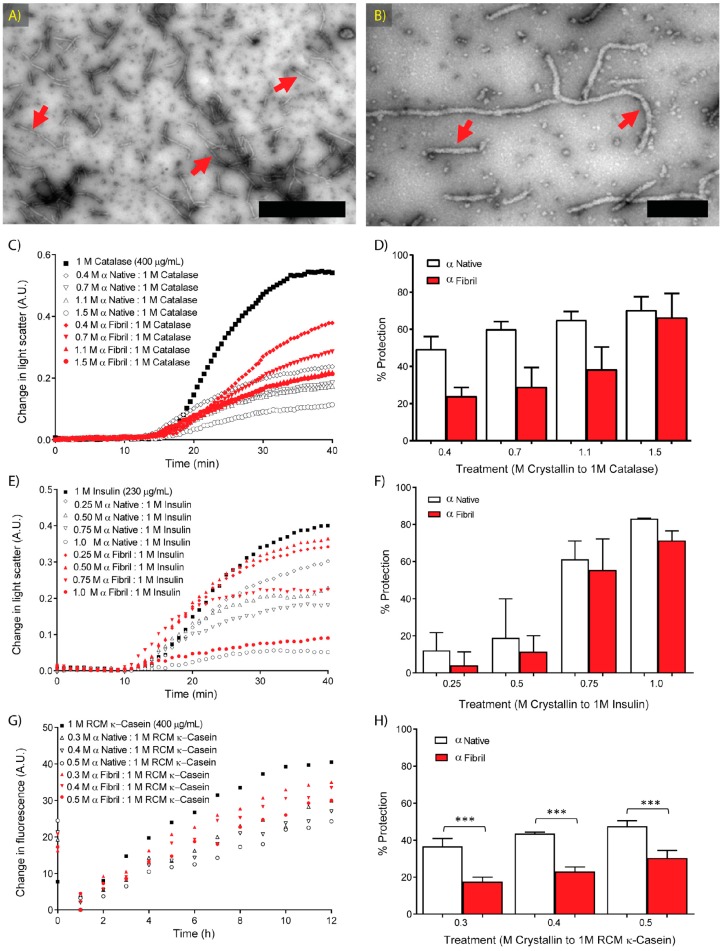
Fibril formation and chaperone activity of native and fibrillar α-crystallin. (**A**,**B**) Transmission electron micrographs of α-crystallin fibrils formed in 1 M guanidine hydrochloride (GdnHCl), 0.1 M sodium phosphate, pH 7.4 at 60 °C for 2 h, with red arrows indicating fibril structures. Scale bars are: (**A**) 1 μm and (**B**) 200 nm; (**C**–**H**) Native and fibrillar α-crystallin chaperone protection of (**C**,**D**) catalase at 400 μg/mL in 0.1 M sodium phosphate buffer at pH 7.4, undergoing thermal destabilisation at 60 °C; (**E**,**F**) reduced insulin, 230 μg/mL in 0.1 M sodium phosphate buffer pH 7.4, at 37 °C in the presence of 10 mM 1,4-dithiothreitol (DTT); and (**G**,**H**) reduced and carboxymethylated (RCM) κ-casein, 400 μg/mL incubated at 37 °C in 0.1 M sodium phosphate, 10 μM thioflavin T (ThT) for 12 h; (**C**,**E**,**G**) show representative profiles of light scattering; (**D**,**F**,**H**) show the percentage of protection provided by each chaperone, calculated from the difference between the maximum light scattering or fluorescence of the target protein alone and the target protein in the presence of the stated concentrations of α-crystallin. Data shown are means ± standard error (SE) of (**D**,**F**) three or (**H**) of six separate experiments; *p-*values indicating significant difference from two-way ANOVA (Šidák post-hoc test) are: *** p < 0.001.

α-crystallin (comprised of ~3:1 molar ratio of αA- to αB-crystallin) was extracted from bovine lenses and purified as described by Chiou et al. (1979) [45]. α-crystallin amyloid fibrils were formed by partial unfolding in denaturant at elevated temperature [4,46] and the transformation from native to fibrillar state was confirmed by transmission electron microcopy (TEM) (Figure 1A,B). The fibrils ranged in length from 20 nm to 1 μM and showed the characteristic morphologies previously described for α-crystallin amyloid fibrils [46]. A number of smaller aggregates, either protofibrils or amorphous aggregates, were also present.

### 2.2. Molecular Chaperone Activity of Native and Fibrillar α-Crystallin against Amorphous Aggregation

The chaperone activity of α-crystallin in native and amyloid fibrillar states was measured against two amorphously aggregating target proteins: catalase destabilised by elevated temperature (60 °C) and insulin destabilised by reduction at 37 °C [47]. Amorphous aggregation and amyloid fibril formation occur in three main stages: a phase during which target protein unfolding and association occurs; an exponential stage, where protein aggregates become large enough to scatter light; and a plateau, where target protein aggregation is complete [48]. Protein aggregation was monitored via light scattering at 340 nm. After approximately 40 min of incubation, both insulin and catalase reached a plateau of light scattering, indicative of the completion of aggregation. The protection afforded by the chaperone is apparent from the reduction in light scattering, and was quantified for further statistical analysis using Equation (1). The chaperone activity against the aggregating target proteins catalase and insulin is presented in Figure 1.

Native and fibrillar α-crystallin both demonstrated concentration-dependent chaperone protection against the thermally-induced amorphous aggregation of catalase (Figure 1C,D). At 50 to 200 μg/mL of chaperone (molar ratios between 0.4:1.0 and 1.5:1.0, chaperone:catalase), there was no significant difference between native and fibrillar α-crystallin in protection against heat-induced amorphous aggregation of catalase, e.g., at the highest concentration tested (i.e. a molar ratio of 1.5:1.0 α-crystallin: catalase), a protection of 70 ± 7.2% and 66 ± 13.0% was observed for native and fibrillar α-crystallin, respectively. There was a significant difference between the activity of native and fibrillar α-crystallin over the concentrations tested, with catalase aggregation being inhibited to a greater degree by native α-crystallin than fibrillar α-crystallin. Both native and fibrillar α-crystallin inhibited the reduction-induced amorphous aggregation of insulin in a comparable, and concentration-dependent manner (Figure 1E,F). In preventing insulin from aggregating, there was no statistical difference between the protection provided by equivalent concentrations of native and fibrillar α-crystallin, e.g., at a 1.0:1.0 molar ratio of α-crystallin to insulin, native and fibrillar α-crystallin inhibited insulin aggregation by 83 ± 0.3% and 71 ± 5%, respectively.

### 2.3. Molecular Chaperone Activity of Native and Fibrillar α-Crystallin against κ-Casein Fibrillar Aggregation

To investigate further this potentially stress-dependent variation in chaperone activity, a third target protein was examined. The milk protein κ-casein has a high propensity to form amyloid fibrils when isolated from the other casein proteins [49,50]. The reduced and carboxymethylated (RCM) form of κ-casein aggregates in a highly reproducible manner, leading to formation of amyloid fibrils at physiological temperature and pH [50,51]. Both native and fibrillar α-crystallin showed a concentration-dependent chaperone activity with regards to inhibiting fibril formation by RCM κ-casein (Figure 1G,H). Against this target protein, significant differences in chaperone ability were observed between the native and fibrillar forms of α-crystallin at each concentration tested. Fibrillar α-crystallin was between 50% to 65% as effective at inhibiting RCM κ-casein fibril formation compared to native α-crystallin, at each of the three molar ratios tested (0.3–0.5:1.0, α-crystallin: RCM κ-casein, respectively). Thus, the fibrillar form of α-crystallin possesses chaperone activity comparable to that of native α-crystallin for the two amorphously aggregating targets assayed. However, with regards to the fibril-forming target protein, RCM κ-casein, the chaperone activity of fibrillar α-crystallin was less than that of native α-crystallin, but it still protected against protein fibril formation in a concentration-dependent manner.

### 2.4. Molecular Chaperone Activity of Native and Fibrillar α-Crystallin as Compared to Non-Chaperone Based Amorphous and Fibrillar Aggregates

**Figure 2:**
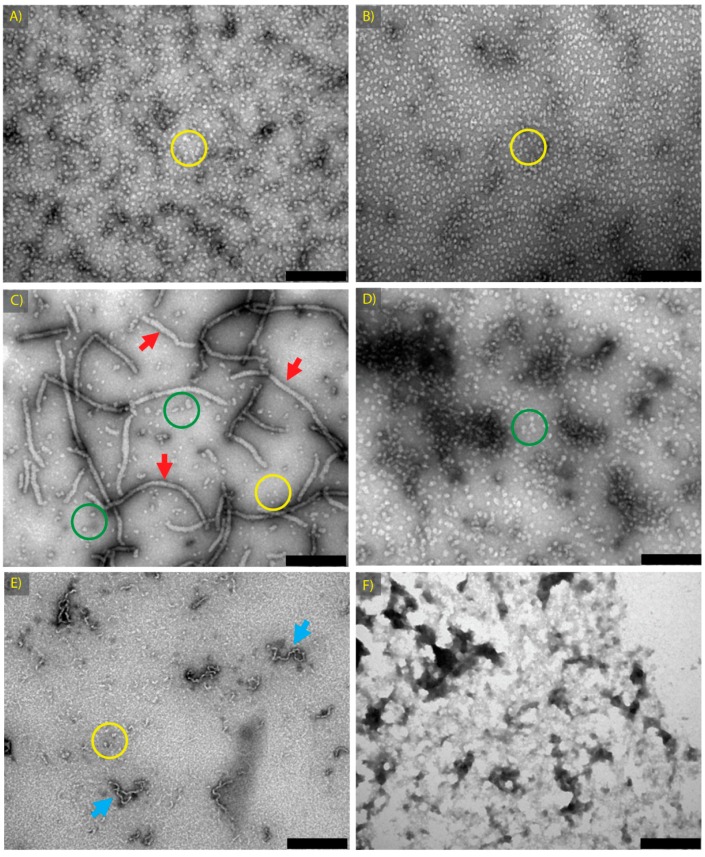
Transmission electron microscopy (TEM) images of samples formed to assess the effects of structural variation on the chaperone activity of α-crystallin: (**A**) α Native; (**B**) α GdnHCl Native; (**C**) α Fibril; (**D**) α Amorphous; samples of α-crystallin. (**E**) βH Fibril; and (**F**) aldehyde dehydrogenase (ADH) Amorphous samples. All samples were prepared as described in Table 1. Scale bars are 200 nm. Features of importance are highlighted, including long amyloid fibrils (red arrows), curvilinear amyloid fibrils (blue arrows) and spherical aggregates of ~15 nm in diameter (yellow circles) or larger spherical aggregates (green circles).

In order to further investigate the chaperone ability of destabilised α-crystallin, a stock of native α-crystallin was prepared with the equivalent concentration of GdnHCl (1 M) used to prepare fibrillar α-crystallin. This stock was stored at 4 °C for use in chaperone assays (‘α GdnHCl Native’) and for fibril preparation (‘α Fibril’). TEM examination showed no observable difference in morphology between native α-crystallin and GdnHCl-treated native α-crystallin, i.e., both appeared as roughly spherical oligomers of approximately 15 nm in diameter (Figure 2A,B, oligomers highlighted with yellow circles), and similar to what has been observed previously [52].

Following sample incubation under conditions conducive for amyloid fibril formation, all samples were spin-filtered to remove any GdnHCl (where present). α-Crystallin amyloid fibrils appeared no different in morphology after spin filtering and were highly comparable to non-spun α-crystallin fibrils in length and concentration (Figure 1B and Figure 2C, amyloid fibrils indicated by red arrows). These structures have been previously characterised by our laboratory as amyloid in nature as they exhibit thioflavin T and Congo red binding, and give a X-ray diffraction pattern characteristic of the highly β-sheeted amyloid fibril structure [4]. In addition to the fibrillar structures, some small spherical oligomers were present (yellow circles) as well as some large, mostly spherical aggregates (green circles).

To examine whether the chaperone activity of aggregated α-crystallin was specific for the fibrillar form, an amorphously aggregated form of α-crystallin was also prepared by incubation in 0.1 M sodium phosphate buffer with 1 M GdnHCl at 90 °C for two hours (‘α Amorphous’). By TEM, this species formed larger, mostly spherical, aggregates with some clumps of protein present (Figure 2D, green circles), a morphology that is consistent with the findings of Burgio et al. [29] and previous findings of our laboratory [53] where an increased size of α-crystallin oligomers at high temperatures was demonstrated, with a tendency for those oligomers to clump into beads or strings.

To test whether chaperone activity was specific to α-crystallin amyloid fibrils, amorphous aggregates and amyloid fibrils were prepared from non-chaperone active proteins. Amyloid fibrils were formed from βH-crystallin (‘βH Fibril’) in the presence of trifluoroethanol (TFE) at low pH under thermal stress conditions [4]. These fibrils are short and curly in nature, as seen in Figure 2E (highlighted with blue arrows). Amorphous aggregates of aldehyde dehydrogenase (ADH) were prepared in 0.1 M sodium phosphate, pH 7.4 with 1 M GdnHCl at 90 °C for 2 h (‘ADH Amorphous’), and appeared as non-fibrillar amorphous protein clumps (Figure 2F).

**Figure 3:**
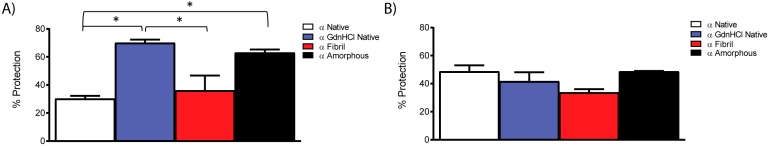
Chaperone protection provided by native, fibrillar and amorphous α-crystallin species against the (**A**) amorphous aggregation of reduced insulin, 250 μg/mL in 0.1 M sodium phosphate, pH 7.4 and 20 mM DTT at 37 °C (0.9 chaperone: 1.0 insulin on a molar basis); and (**B**) fibrillar aggregation of RCM-κ-casein, 400 μg/mL incubated at 37 °C for 22 h in the presence of various native, amorphous and fibrillar chaperone species (0.5 chaperone: 1.0 RCM κ-casein on a molar basis) and monitored via ThT fluorescence. The percentage of protection provided by each chaperone is calculated from the difference between the maximal light scattering or fluorescence of the target protein alone and the target protein in the presence of the stated concentrations of α-crystallin. Results are mean ± SE of the percentage protection given by chaperones for three experiments; *p-*values, derived by one-way ANOVA with Tukey post-test, are * *p* < 0.05.

Table 1 outlines the conditions used to prepare these protein species and the morphology of each as observed by TEM. All samples, following incubation (at 4, 60 or 90 °C), were dialysed to remove GdnHCl prior to assaying for chaperone activity. For ease of reference, the nomenclature in Table 1 is used throughout this work. Within one hour of preparation, samples were used for chaperone activity assays against insulin as a model of amorphous aggregation and RCM κ-casein as a model of fibrillar aggregation (Figure 3). The assays highlight the ability of α-crystallin to act as a chaperone under a range of conditions.

Both α Native and α Fibril exhibited equivalent chaperone activity in preventing the reduction-induced aggregation of insulin (Figure 3A). The overall chaperone activity for all treatments was consistently lower compared to earlier assays, most likely as a result of protein loss during the spin filter process (used for all samples to remove GdnHCl where present). α GdnHCl Native and α Amorphous both had enhanced chaperone activity compared to α Native, whereas both βH Fibril and ADH Amorphous species demonstrated no chaperone activity (Figure S1A). α Amorphous exhibited enhanced chaperone activity compared to α Native, which may be responsible for the chaperone activity of the α Fibril sample, as it contained both amyloid fibrils and amorphous aggregates (Figure 2C). The enhanced activity of α-crystallin in the presence of GdnHCl has been demonstrated previously [26], where an intermediate structure was proposed to have enhanced chaperone activity. Furthermore, after urea denaturation, α-crystallin can refold into oligomers with increased chaperone activity, coincident with an increase in surface exposed hydrophobicity [30].

When investigating inhibition of the amyloid fibril formation of RCM κ-casein, all native and aggregated α-crystallin species (α Native, α GdnHCl Native, α Fibril and α Amorphous) acted with similar chaperone efficacy (Figure 3B). In contrast, there was no chaperone activity exhibited by aggregates of non-chaperone proteins, including βH Fibril and ADH Amorphous (Figure S1B). In fact, both non-chaperone species caused a slight increase in ThT fluorescence associated with fibril formation, possibly through a molecular crowding effect, with the presence of additional protein promoting amyloid fibril assembly by enhancing self-association. Thus, all α-crystallin species (native, previously destabilised, amyloid fibrillar and amorphous aggregates) retain chaperone activity against RCM κ-casein aggregation.

Unlike the activity of α-crystallin species against amorphous insulin aggregation, there was no significantly enhanced protection provided by amorphous aggregates of α-crystallin, making it difficult to determine if the chaperone activity of α Fibril is the effect of the amyloid fibrils themselves or of the amorphous aggregates present in this sample (or a combination of both). As observed with amorphous aggregation at 37 °C, there was no chaperone activity from non-chaperone aggregates (fibrillar or amorphous, Figure S1B), indicating that the chaperone activity exhibited by aggregated α-crystallin species is not due to their exposed hydrophobicity.

The ability of destabilised and amorphous aggregates of α-crystallin to act as chaperones makes it unclear whether α-crystallin fibrils actually retain chaperone activity. It is possible that the observed chaperone activity of samples containing α-crystallin fibrils is due to the heightened activity (in some cases, for example against reduced insulin) of α-crystallin amorphous aggregates which are also present within these samples (Figure 2C). While the ability of amorphous aggregates of α-crystallin to act effectively as a molecular chaperone is of interest in itself, and will be discussed in detail below, a clearer understanding of whether α-crystallin amyloid fibrils can act as molecular chaperones is desired, hence a study of the chaperone ability of αB-crystallin was undertaken.

### 2.5. Chaperone Activity of αB-Crystallin Fibrils and Other Species

αB-crystallin shows similar chaperone efficaciousness to α-crystallin in vitro at 37 °C. Previous work in our laboratory has shown that amyloid fibrils, as defined by the X-ray fibre diffraction pattern, formed from recombinant αB-crystallin contain less than 5% amorphous aggregates, i.e., much less than is present in α-crystallin fibrillar samples [46]. Thus, αB-crystallin was selected for use in the same assays to those already performed on α-crystallin. The higher purity of αB-crystallin fibrils arises from less native protein and fewer amorphous aggregates, which enables simpler interpretation of the effects of fibril formation on the chaperone activity of αB-crystallin. For comparison and as controls, identical samples to those assessed for α-crystallin were prepared from αB-crystallin, i.e., αB Native; αB GdnHCl Native; αB Fibril; and αB Amorphous, as were the non-α-crystallin controls βH Fibril; and ADH Amorphous (Table 1).

**Figure 4:**
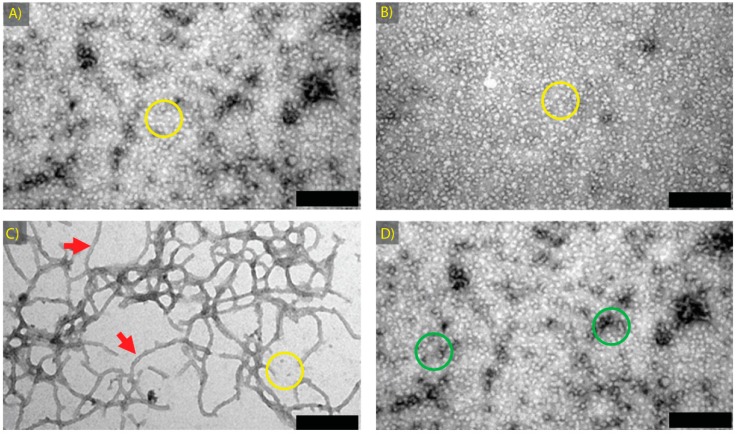
TEM images of αB-crystallin species formed to assess the effects of structural variation on chaperone activity: (**A**) αB Native; (**B**) αB GdnHCl Native; (**C**) αB Fibril; and (**D**) αB Amorphous. All samples were prepared as described in Table 1. Scale bars are 200 nm. Features of importance are highlighted, including long amyloid fibrils (red arrows) and spherical aggregates of ~15 nm in diameter (yellow circles) or larger spherical aggregates (green circles).

Spin filtering to remove GdnHCl (where present) was again performed on all samples. All species were then examined by TEM prior to chaperone assessment (Figure 4). As before, fibril structures are shown with red arrows and aggregates are highlighted with yellow and green circles. αB-crystallin species were almost identical in appearance to those of α-crystallin (Figure 2 and Figure 4), with the exception of the αB Fibril sample, which featured amyloid fibrils over 1 μm in length with almost no amorphous aggregates present (Figure 4C). There were visibly fewer amorphous structures present in the αB Fibril sample when compared to the α Fibril sample (Figure 2C and Figure 4C), again consistent with what has been observed previously [4,46]. The chaperone ability of these species was assessed against the reduction-induced amorphous aggregation of insulin and RCM κ-casein amyloid fibril aggregation, both at 37 °C.

**Figure 5:**
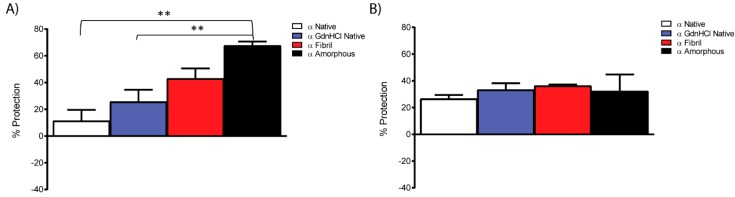
Chaperone protection provided by native, fibrillar and amorphous αB-crystallin species against the (**A**) amorphous aggregation of insulin, 250 μg/mL at 37 °C in 0.1 M sodium phosphate, 20 mM DTT, pH 7.4 (0.9 chaperone: 1.0 insulin on a molar basis); and (**B**) fibrillar aggregation of RCM κ-casein 400 μg/mL, 10 μM ThT at 37 °C, pH 7.4 (0.5 chaperone: 1.0 RCM κ-casein on a molar basis). The percentage of protection provided by each chaperone is calculated from the difference between the maximal light scattering or fluorescence of the target protein alone and the target protein in the presence of the stated concentrations of αB-crystallin. Results are mean ± SE of the percentage protection given by chaperones for three experiments; *p*-values, derived by one-way ANOVA with Tukey post-test, are ** *p* < 0.01.

Destabilised forms of αB-crystallin, including the GdnHCl-treated native protein and both fibrillar and amorphous aggregates, prevented both insulin amorphous aggregation and RCM κ-casein amyloid fibril formation, as summarised in Figure 5. For both target proteins, there was no statistically significant difference in chaperone activity of αB Fibril and αB Native. As observed for α-crystallin amorphous aggregates, the αB Amorphous sample was significantly more effective against insulin amorphous aggregation (over three times as effective as αB Native, Figure 5A), yet had equivalent activity to native αB-crystallin with regards to inhibiting RCM κ-casein amyloid fibril formation (Figure 5B). The increased ability of amorphous α-crystallin samples to inhibit insulin aggregation thus appears to be specific to that target protein. Furthermore, it is unlikely to be an effect of enhanced exposed hydrophobicity of this species, as ADH amorphous aggregates and βH crystallin amyloid fibrils exhibited no chaperone activity against these target proteins (Figure S2).

## 3. Discussion

It is well recognised that α-crystallin maintains chaperone activity even under conditions of structural change. Thus, the chaperone ability of α-crystallin is enhanced when it is structurally perturbed or partially unfolded, i.e., under conditions of increased temperature or GdnHCl exposure [24,25,26,54,55]. Furthermore, a range of other treatments and conditions enhance the chaperone activity of α-crystallin, including elevated pressure, the presence of various small molecules and metal ions, and some post-translational modifications, for example the mimicking of phosphorylation [47,56,57,58,59]. Remarkably, α-crystallin retains some of its chaperone activity even when dissociated and severely structurally destabilised at low pH [60]. The work presented herein demonstrates that, even after undergoing major structural rearrangement into amyloid fibrils or amorphous aggregates, α-crystallin retains significant chaperone activity. Consistent with these results, the high-molecular weight (HMW) form of α-crystallin isolated from aged bovine lenses, which has an altered structure compared to the native form of the protein, retains some chaperone activity [44].

The chaperone activity of α-crystallin species was compared with various aggregated control proteins, with regards to its capacity to inhibit the reduction-induced amorphous aggregation of insulin and fibrillar aggregation of RCM κ-casein. In both cases, the α-crystallin species retained chaperone activity whilst non-chaperone based amyloid fibrils and amorphous aggregates provided no protection to the aggregating proteins. It is extremely unlikely, therefore, that the observed chaperone activity is due to generic chaperone activity of exposed hydrophobic regions on fibrils or amorphous aggregates. It is possible that some of the observed chaperone action arises from dissociated forms of α-crystallin. However, we contend that the dissociated species cannot be responsible for all the chaperone activity of the fibrillar α-crystallin samples. We base this on two factors: first, the majority of species in the α Fibril sample, and the vast majority of the species in the αB Fibril sample, are in the fibrillar form (as is apparent from the TEM images); and second, the extent of the chaperone action, which in some cases is similar to that of the native species. The combination of these factors argues against dissociated forms of α-crystallin being solely responsible for the observed chaperone action. Instead, it is concluded that part, if not all, of the chaperone ability of amyloid fibrillar and amorphous aggregates of α-crystallin, is due to the chaperone activity of α-crystallin being retained upon aggregation to different structural forms. This is consistent with previous findings from our laboratory, which demonstrated that α-crystallin immobilised onto a solid-phase support (i.e., when no dissociated species were present) had enhanced chaperone ability compared to native α-crystallin [61]. Thus, for α-crystallin and αB-crystallin, the structural changes that occur in amyloid fibril formation do not necessarily result in a loss of chaperone function.

The ability of fibrillar αB-crystallin to act as a molecular chaperone has in vivo relevance, both within and outside the lens where αB-crystallin is found extensively. The conversion into amyloid fibrils of a protein from its native state (whether structured or not) requires a dramatic alteration in structure, and conceptually, it would be considered unlikely that biological function is retained after such structural perturbation. In a related context, functional amyloid comprises protein aggregates which exhibit biological activity whilst adopting the cross β-sheet structure characteristic of amyloid fibrils [36,41,62,63]. Unlike α-crystallin fibrils, which occur as a mixed population of amyloid fibrils and amorphous aggregates, αB-crystallin amyloid fibrils are formed with very few amorphous aggregates present. Furthermore, against RCM κ-casein amyloid fibril formation, αB-crystallin amyloid fibrils acted with enhanced activity, while amorphous aggregates of αB-crystallin had equivalent chaperone ability to native αB-crystallin.

Significant parts of the amino acid sequences of both αA-crystallin and αB-crystallin are potentially amyloidogenic [15]. They are primarily located in the central, conserved β-sheet-rich α-crystallin domain (ACD) and are absent in the unstructured and relatively flexible C-terminal region. The N-terminal region that flanks the ACD is also relatively unstructured, and has very few regions of potential propensity to form amyloid fibrils. The C-terminal region, particularly the highly flexible last 10 to 12 amino acids in both subunits, plays a role in solubilising the protein and both the N- and C-terminal regions mediate interactions between the chaperone protein and its targets during chaperone action [10,15,27]. Notably, the region of αA-crystallin encompassing residues 71 to 88 in the ACD is both chaperone active as an isolated peptide and highly amyloidogenic [64]. The peptide loses its fibril-forming propensity, whilst maintaining chaperone activity, when coupled to the highly flexible C-terminal extension of αA-crystallin [64]. Conceivably, the similar properties that govern the interaction and binding of α-crystallin to an unfolding target protein during chaperone action are present during the mutual interaction to form fibrillar aggregates. The flexible C-terminal extension of αB-crystallin is not incorporated into the fibril core; it maintains its intrinsic disorder [46] and as such is available to mediate interactions between aggregated, fibrillar α-crystallin and other proteins.

The lens is a unique organ; it continues to grow throughout life yet lacks a blood supply and exhibits no protein turnover. As such, the crystallin proteins in its centre are as old as the individual [1,2]. To maintain lens transparency and the high refractive index necessary for vision, the crystallins must remain stable and in solution. With age, extensive modification occurs to the crystallins, i.e., deamidation, racemisation, phosphorylation, truncation, glycation etc. which collectively affect their structure, solubility and potentially lead to precipitation and lens opacification and cataract formation. The two α-crystallin subunits are particularly subjected to modification and large-scale aggregation into HMW species. In fact, there is little, if any, intact α-crystallin remaining in the normal, transparent human lens after 40 years of age [44,65]. Thus, our observation of the retention of chaperone activity in heavily structurally modified (including amyloid fibrillar) α-crystallin is consistent with the in vivo behaviour of modified α-crystallin in older, non-cataract (transparent) lenses, in being a functioning chaperone to prevent crystallin aggregation [44]. It is also compatible with the notion that aged α-crystallin in the lens may adopt an amyloid fibrillar structure [4,46], in addition to forming amorphous aggregates, and that both retain some chaperone ability.

## 4. Materials and Methods

### 4.1. Materials

Alcohol dehydrogenase (ADH), κ-casein, catalase and insulin were obtained from Sigma Chemical Co. (St. Louis, MO, USA). 1,4-dithiothreitol (DTT) and thioflavin T (ThT), were also purchased from Sigma. All other chemical reagents were purchased from AJAX Finechem (Cheltenham, VIC, Australia). Clear and μClear 96-well plates were obtained from Greiner Bio-One (Kremsmünster, Austria). ThinSeal was purchased from Excel Scientific Inc. (Victorville, CA, USA). Supor (0.2 μm) syringe filters and Macropep 10 kDa spin tubes were obtained from Pall Life Sciences (Port Washington, NY, USA). Formvar and carbon coated nickel electron microscopy grids were purchased from Pro Sci Tech (Kirwan, QLD, Australia).

Bovine eye lenses were supplied by local abattoirs (T. & R. Pty. Ltd., Murray Bridge, South Australia and CMP Canterbury Ltd., Ashburton, New Zealand). *Escherichia coli* containing the plasmid vector pET20b(+) with the human wild type αB-crystallin gene previously inserted was obtained from Prof. Wilbert Boelens, Radboud University, The Netherlands.

### 4.2. Methods

#### 4.2.1. Bovine Crystallin Protein Extraction

Bovine crystallin proteins were extracted using standard methods, adapted from Carver et al. [44]. Multiple lenses were homogenised using a manual homogeniser in 3 mL buffer per lens, with the buffer containing 50 mM Tris pH 7.2, 1 mM DTT and 0.04% NaN_3_. The homogenate was centrifuged at 13,000× *g* for 30 min at room temperature. The supernatant, or crude crystallin stock, was stored at −20 °C.

#### 4.2.2. Bovine Crystallin Separation

Extracted crystallin proteins were separated using size exclusion chromatography (SEC). Separation was performed using an AKTA FPLC (Amersham Biosciences, Little Chalfont, UK). Aliquots of homogenate (1 mL) were run over a Sephacryl 300 Highprep 26/60 column (Amersham Biosciences). The composition of semi-purified crystallin stocks was assessed using sodium dodecyl sulfate polyacrylamide gel electrophoresis (SDS-PAGE) with a wide range marker (Sigma) and only non-contaminated samples used for further assays.

#### 4.2.3. Expression and Purification of Human αB-Crystallin

Protein expression was initiated by plating and streaking out a small amount of thawed vector-containing *E. coli* onto an ampicillin imbued agar plate. The culture plate was incubated at 37 °C overnight to allow colonies to develop. Once colonies over the size of 2 mm were present, the plate was placed to 4 °C and stored for up to two weeks. Only individual colonies were selected for further culture and large-scale protein expression.

Large scale protein expression, also following the methods of [48], involved the transfer of an individual *E. coli* colony (containing the plasmid vector) to 50 mL lysogeny broth (LB) media with ampicillin (50 ng ampicillin:1 mL), culture was then grown at 37 °C with shaking overnight. The cultured media was then distributed between 2 L of LB media (without ampicillin) and culturing at 37 °C with shaking continued. Once the *E. coli* had grown adequately, with an optical density at 600 nm between 0.6 and 1.0, protein expression was induced by the addition of 0.25 mM isopropyl β-D-1-thiogalactopyranoside. The *E. coli* was incubated for a further three to four hours (as above) to allow protein expression to occur. Culture flasks were then removed from the incubator and the cells were centrifuged at 5000× *g* for 20 min to form a cell pellet. The supernatant was discarded and the cell pellet collected, weighed and stored at −20 °C until cell lysis and protein purification (a maximum of one month).

The *E. coli* cell pellet was defrosted by resuspension in an ice-cold buffer of 50 mM Tris and 100 mM NaCl at pH 8, using 3 mL of buffer per gram of cell pellet (gcp). To this was added phenylmethane sulfonyl fluoride (2 μL of 0.5 M stock per gcp), lysozyme (0.8 mg per gcp) and deoxycholic acid (4 mg per gcp). The cell lysis mixture was then placed at room temperature and gently shaken for 45 min. DNA was broken down by addition of DNAse I (2 μL of a 10 mg/mL stock per gcp) with shaking at room temperature until the mixture lost viscosity. Metals were removed by the addition of ethylenediaminetetraacetic acid (EDTA) (8 μL of 250 mM stock per gcp) and the waste components removed by centrifugation at 12,000× *g* for 30 min at 4 °C. The un-purified protein fraction, contained in the supernatant, was collected.

Purification of recombinant human αB-crystallin from the cell lysis supernatant was achieved using anion exchange chromatography. Five mg of DTT and 36 μL of polyethylenimine per gcp were added to the cell lysis supernatant and incubated at room temperature with constant mixing for 20 min. Waste proteins were removed by centrifugation at 13,000× *g* for 30 min at 4 °C, with the αB-crystallin containing supernatant collected and loaded onto a diethylaminoethyl (DEAE) column (XK 26, packed with Sepharose Fast Flow gel, Amersham Biosciences) which had been equilibrated with three column volumes of 20 mM Tris, 1 mM EDTA at pH 8. αB-crystallin was removed via a salt gradient using a buffer of 20 mM Tris, 1 mM EDTA and 1 M NaCl. αB-crystallin was the primary peak eluted and contained a pure band of an approximately 20 kDa protein. Protein purity was assessed by SDS-PAGE.

#### 4.2.4. Aggregate Formation for Use as Molecular Chaperones

##### Refolded Aggregate Formation for Use as Molecular Chaperones

α- and αB-crystallin refolded oligomers were formed to examine their molecular chaperone ability. 3 mg/mL of each protein was reconstituted into 0.1 M sodium phosphate, 1 M GdnHCl and incubated at 4 °C for two hours. To allow refolding of subunits the removal of GdnHCl was achieved as outlined below.

##### Amorphous Aggregate Formation for Use as Molecular Chaperones

Three mg/mL each of α- and αB-crystallin was reconstituted into 0.1 M sodium phosphate, 1 M GdnHCl and incubated at 90 °C for two hours. Where required, samples were treated using the method for removal of GdnHCl (see below) to produce equivalent conditions and thus allow for comparison of samples.

##### Amyloid Fibril Formation by α-Crystallin Using GdnHCl

α- and αB-crystallin amyloid fibrils were formed using methods adapted from Meehan et al. (2004) [4]. Three mg/mL of each protein was reconstituted into 0.1 M sodium phosphate, 1 M GdnHCl and incubated at 60 °C for two hours. Where required, samples were treated using the method for removal of GdnHCl (see below) to produce equivalent conditions and thus allow for comparison of samples.

#### 4.2.5. Monitoring Protein Aggregation

##### Induction of Amorphous Aggregation

Amorphous aggregation of the target protein was induced in two ways, either by temperature destabilisation or by the reduction of disulfide bonds [58,66]. Protein precipitation increases the turbidity of solutions which was monitored by light scattering at a wavelength of 340 nm [58]. Data were acquired using a Fluostar Optima plate reader (BMG Lab technologies, Mornington, VIC, Australia). In this work, all proteins (target and chaperone) were prepared in 0.1 M sodium phosphate at pH 7.4 and filtered with 0.2 μm filter immediately prior to further sample preparation (either formation into amorphous or fibrillar aggregates). Assays were run in clear 96-well plates, using a sample volume of 200 μL/well. No agitation of solutions was used.

##### Light Scattering Assessment of Thermally Induced Amorphous Aggregation

Catalase was used to assess the ability to inhibit temperature-induced (in this case 60 °C) amorphous aggregation. Assay samples included: buffer alone (0.1 M sodium phosphate at pH 7.4); catalase in buffer; chaperone protein in buffer; and catalase and chaperone protein in buffer. All samples were run in duplicate, and the activity of each chaperone protein assessed over three to six separate experiments. Aggregation was determined as maximum change in light scattering at 340 nm ± 10 nm between the initial time-point and the final time-point. Final time-points were determined by a plateau in light scattering (i.e., completion of protein precipitation by catalase at 40 min). Results displayed are the mean ± standard error (SE) for all replicates.

##### Light Scattering Assessment of Disulfide-Bond Reduction Induced Amorphous Aggregation

Insulin was used to assess the ability to inhibit the reduction-induced amorphous aggregation of a target protein. Assay samples included: buffer with DTT (0.1 M sodium phosphate at pH 7.4, 20 mM DTT); insulin in buffer with DTT; chaperone protein in buffer with DTT; and insulin and chaperone protein in buffer with DTT. Assays were performed at 37 °C Aggregation was assessed as per the catalase aggregation assays, with a plateau in light scattering at 40 min for all assays. Results displayed are the mean ± SE for all replicates.

#### 4.2.6. Characterisation of Amyloid Fibril Formation

##### Amyloid Fibril Formation by RCM κ-Casein

RCM κ-casein was reconstituted in 0.1 M sodium phosphate and 10 μM ThT, pH 7.4. Amyloid fibrils were formed upon incubation at 37 °C with linear shaking at 115 rpm for up to 20 h, following the method of Thorn et al. (2005) [50].

##### Thioflavin T Assay of Amyloid Fibril Formation

ThT assays were performed in situ by a method adapted from Nielsen et al. (2001) [67]. Samples were incubated with 10 μM ThT in μClear 96-microwell plates using a sample volume of 100–200 μL per well. Plates were sealed with ThinSeal (Excel Scientific Inc., Victorville CA, USA) to prevent evaporation, and incubated at 37 °C with or without shaking. Fluorescence was measured at regular intervals using Fluostar Optima plate reader (BMG Lab technologies, Australia) with a 440/490 nm excitation/emission filter set (±10 nm) until a plateau in fluorescence was obtained (different for each target protein). All proteins (target and chaperone) were prepared in 0.1 M sodium phosphate at pH 7.4 and filtered with 0.2 μm filter immediately prior to use (i.e., prior to formation into potential chaperone structures). Sample volume was 200 μL/well and no agitation of solutions was used. To allow comparison between increases in ThT fluorescence, each curve was normalised relative to the initial fluorescence intensity (change in fluorescence) to eliminate artefacts resulting from the ThT binding propensity of the native target peptide or protein. The maximum change in fluorescence (between the initial time-point and the final time-point) was used for all further calculations, and the mean ± SE of three replicates was used for all statistics and graphs.

##### Transmission Electron Microscopy

TEM was performed as described by Thorn et al. [50]. Samples were prepared on formvar and carbon-coated nickel electron microscopy grids. Protein samples were diluted to 0.5 or 1 mg/mL and 2 μL was deposited on each grid. The grid was rinsed three times with 10 μL of H_2_O, negatively stained with 10 μL of uranyl acetate (2% *w*/*v*) and dried with filter paper. Samples were analysed under magnifications of 20,000× to 130,000×, using an excitation voltage of 120 kV on a Philips Technai 100 transmission electron microscope (FEI, Hillsborough, OR, USA). For all samples, multiple regions (at least three) on the grid were examined to assess structure dispersal. For initial nanofibre assessment, at least three separate samples were examined. For monitoring of chaperone activity against known amyloid or amyloid-like fibril forming proteins, TEM assessment was performed on target protein alone and target protein in the presence of chaperone for three separate experiments.

##### Removal of GdnHCl from α-Crystallin Samples for Use as Molecular Chaperones

To remove GdnHCl from solutions of α-crystallin aggregate species, native protein (GdnHCl-free control), GdnHCl unfolded protein, amorphous aggregates or amyloid fibrils were spun at 14,000× *g* in Macropep 10 kDa spin tubes (Merck Millipore, Bayswater, VIC, Australia) for three consecutive 30 min washes (with 0.1 M sodium phosphate buffer, pH 7.4) and then resuspended into an equivalent volume of 0.1 M sodium phosphate buffer, pH 7.4.

##### Quantification of Molecular Chaperone Activity

Chaperone activity was determined from the light scattering and ThT variables measured as described above. The percentage of protection provided by a given sample is derived from the difference between the maximum change in light scattering or ThT fluorescence for the target protein alone and in the presence of the chaperone, i.e., the maximum change in light scattering or ThT fluorescence for the target protein is judged to be 100% aggregated, upon addition of an active chaperone to the target protein a drop in maximum light scattering or ThT fluorescence would be observed, this equates to less target protein aggregated. The following equation (Equation (1)) [58] was used to quantify the protection provided by each chaperone:

$$\mathrm{Percentage}\;\mathrm{Protection}=\left(\frac{Max_{\mathrm{Target}\;\mathrm{Protein}}-Max_{\mathrm{Target}\;\mathrm{Protein}+\mathrm{Chaperone}}}{Max_{\mathrm{Target}\;\mathrm{Protein}}}\right)\times 100$$

where *Max* is the maximal change observed in light scattering or ThT fluorescence between the initial time-point and end time-point of the assay. All statistical analysis of percent protection results was performed on the mean percent protection value of three to six replicates. Data are presented as mean ± SE where *p* < 0.05 was considered significant.

## 5. Conclusions

For over 25 years, α-crystallin has been recognised as a molecular chaperone of importance in the prevention of cataract and other protein aggregation disorders [9,48]. The primary role of α-crystallin as a molecular chaperone is to inhibit protein aggregation under conditions of cellular stress. As a result, the ability of α-crystallin to function effectively as a chaperone when structurally perturbed is a desirable biological quality. In this work, it has been established that both α-crystallin and αB-crystallin retain chaperone activity following destabilisation by GdnHCl, and when forming amorphous aggregates and amyloid fibrils. Indeed, αB-crystallin has enhanced chaperone activity after modification into amyloid fibrils. Thus, α-crystallin’s role as a molecular chaperone in vivo is likely to be maintained even under conditions where it does not sustain its native conformation, and is converted into other structural forms, including amyloid fibrils. Indeed, the structured ACD region of αB-crystallin on its own has significant chaperone ability [68]. Thus, truncation of αB-crystallin termini does not alter chaperone functionality. Accordingly, it may be proposed that nature has engineered α-crystallin to be malleable, both structurally and functionally. When in a fibrillar form, α-crystallin exhibits properties of functional amyloid. In the lens, the retention of chaperone ability of various non-native structural forms of α-crystallin has important implications. With ageing of the lens, where there is no protein turnover, the large number of post-translational modifications that occur to α-crystallin, with concomitant unfolding and potential aggregation (e.g., to form amorphous and/or amyloid fibrils), may not alter significantly its ability to prevent the unfolding and potential aggregation of the other crystallins. Such retention of chaperone function after modification and/or aggregation would serve to protect the eye lens from opacification and cataract formation.

## Figures and Tables

**Table 1:** α- and αB-crystallin species formed for chaperone assessment of structural variants.

Treatment	Protein	Incubation Conditions					
		Buffer	pH	Temp. (°C)	Time (h)	Morphology	Figure
α Native	α-Crystallin	0.1 M sodium phosphate	7.4	4	2	Spherical aggregates ~15 nm in diameter	2A
α GdnHCl Native	α-Crystallin	1 M GdnHCl, 0.1 M sodium phosphate	7.4	4	2	Spherical aggregates ~15 nm in diameter	2B
α Fibril	α-Crystallin	1 M GdnHCl, 0.1 M sodium phosphate	7.4	60	2	Long fibrils, (20 nm–1 μm in length), plus short fibrils and/or spherical aggregates	2C
α Amorphous	α-Crystallin	1 M GdnHCl, 0.1 M sodium phosphate	7.4	90	2	Larger spherical aggregates ~30 nm in diameter, singly and in clumps	2D
βH Fibril	βH-Crystallin	H_2_O with 10% TFE	2	60	18	Short, curly fibrils, 20 nm to 200 nm in length	2E
ADH Amorphous	ADH	1 M GdnHCl, 0.1 M sodium phosphate	7.4	90	2	Large non-fibrillar protein clumps	2F
αB Native	αB-Crystallin	0.1 M sodium phosphate	7.4	4	2	Spherical aggregates ~15 nm in diameter	4A
αB GdnHCl Native	αB-Crystallin	1 M GdnHCl, 0.1 M sodium phosphate	7.4	4	2	Spherical aggregates ~15 nm in diameter	4B
αB Fibril	αB-Crystallin	1 M GdnHCl, 0.1 M sodium phosphate	7.4	60	2	Long fibrils, (>1 μm in length)	4C
αB Amorphous	αB-Crystallin	1 M GdnHCl, 0.1 M sodium phosphate	7.4	90	2	Larger spherical aggregates ~30 nm in diameter, singly or in row-like clumps	4D

ADH: aldehyde dehydrogenase; GdnHCl: guanidine hydrochloride; Temp.: temperature; TFE: trifluoroethanol.

## Supplementary Materials

The following are www.mdpi.com/2218-273X/7/3/67/s1, Figure S1: Chaperone protection provided by native α-crystallin compared to non α-crystallin aggregates, Figure S2: Chaperone protection provided by native αB-crystallin compared to non α-crystallin aggregates.
