# Supplementary material for: Functional Amyloid Protection in the Eye Lens: Retention of α-Crystallin Molecular Chaperone Activity after Modification into Amyloid Fibrils
Source: Biomolecules. 2017 Sep 12;7(3):67. doi: 10.3390/biom7030067 (PMC5618248; doi:10.3390/biom7030067)
Supplement: Supplementary file 1 [file biomolecules-07-00067-s001.pdf]

Supplementary Materials

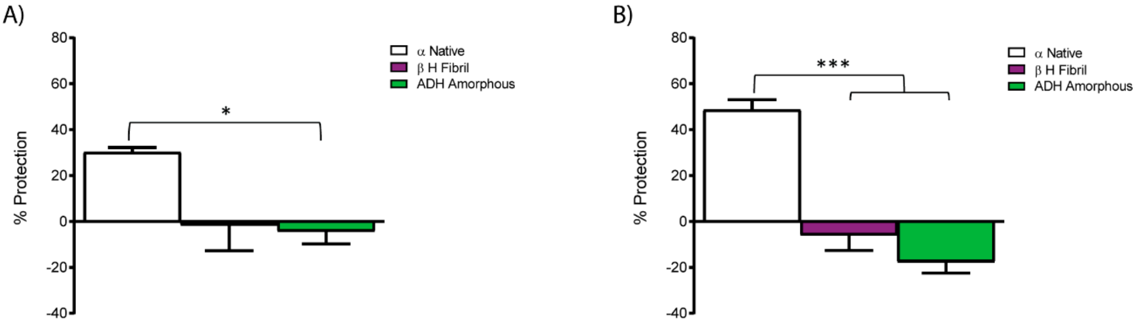

Figure S1. Chaperone protection provided by native α-crystallin compared to non α-crystallin aggregates (both fibrillar and amorphous) against the (**A**) amorphous aggregation of reduced insulin, 250 μg/mL in 0.1 M sodium phosphate, pH 7.4 and 20 mM DTT at 37°C (potential chaperones at 800 μg/mL); and (**B**) fibrillar aggregation of RCM κ-Casein 400 μg/mL incubated at 37 °C for 22 hours (potential chaperones at 200 μg/mL) and monitored via ThT fluorescence. The percentage of protection provided by each chaperone is calculated from the difference between the maximal light scattering or fluorescence of the target protein alone and the target protein in the presence of the stated concentrations of α-crystallin. Results are mean ± SE of the percentage protection given by chaperones for three experiments; *p*-values, derived by one-way ANOVA with Tukey post-test, are * $p < 0.05$, ** $p < 0.01$, *** $p < 0.001$.

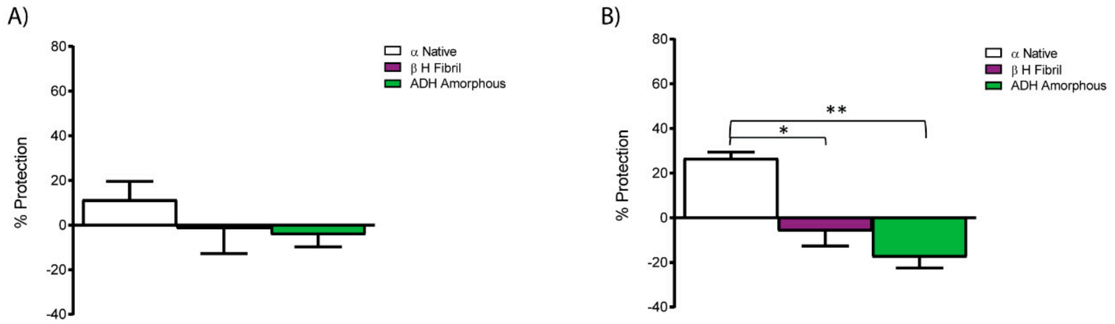

Figure S2. Chaperone protection provided by native αB-crystallin compared to non α-crystallin aggregates (both fibrillar and amorphous) against the (**A**) amorphous aggregation of reduced insulin, 250 μg/mL in 0.1 M sodium phosphate, pH 7.4 and 20 mM DTT at 37°C (potential chaperones at 800 μg/mL); and (**B**) fibrillar aggregation of RCM κ-Casein 400 μg/mL incubated at 37 °C for 22 hours (potential chaperones at 200 μg/mL) and monitored via ThT fluorescence. The percentage of protection provided by each chaperone is calculated from the difference between the maximal light scattering or fluorescence of the target protein alone and the target protein in the presence of the stated concentrations of αB-crystallin. Results are mean ± SE of the percentage protection given by chaperones for three experiments; *p*-values, derived by one-way ANOVA with Tukey post test, are * $p < 0.05$, ** $p < 0.01$, *** $p < 0.001$.
